# Temporal stability in the genetic structure of *Sarcoptes scabiei *under the host-taxon law: empirical evidences from wildlife-derived *Sarcoptes *mite in Asturias, Spain

**DOI:** 10.1186/1756-3305-4-151

**Published:** 2011-07-27

**Authors:** Samer Alasaad, Álvaro Oleaga, Rosa Casais, Luca Rossi, Annarita Molinar Min, Ramón C Soriguer, Christian Gortázar

**Affiliations:** 1 Institute of Evolutionary Biology and Environmental Studies (IEU), University of Zürich, Winterthurerstrasse 190, 8057 Zürich, Switzerland; 2 Estación Biológica de Doñana, Consejo Superior de Investigaciones Científicas (CSIC), Avda. Américo Vespucio s/n 41092 Sevilla, Spain; 3 Instituto de Investigación en Recursos Cinegéticos IREC (CSIC-UCLM-JCCM). Ronda de Toledo, s/n, 13071, Ciudad Real, Spain; 4 SERIDA, Servicio Regional de Investigación y Desarrollo Agroalimentario, Centro de Biotecnología Animal, 33394 Deva-Gijón, Asturias, Spain; 5 Dipartimento di Produzioni Animali, Epidemiologia ed Ecologia, Università degli Studi di Torino, Via Leonardo da Vinci 44, I-10095, Grugliasco, Italy

## Abstract

**Background:**

Implicitly, parasite molecular studies assume temporal genetic stability. In this study we tested, for the first time to our knowledge, the extent of changes in genetic diversity and structure of *Sarcoptes *mite populations from Pyrenean chamois (*Rupicapra pyrenaica*) in Asturias (Spain), using one multiplex of 9 microsatellite markers and *Sarcoptes *samples from sympatric Pyrenean chamois, red deer (*Cervus elaphus*), roe deer (*Capreolus capreolus*) and red fox (*Vulpes vulpes*).

**Results:**

The analysis of an 11-years interval period found little change in the genetic diversity (allelic diversity, and observed and expected heterozygosity). The temporal stability in the genetic diversity was confirmed by population structure analysis, which was not significantly variable over time. Population structure analysis revealed temporal stability in the genetic diversity of *Sarcoptes *mite under the host-taxon law (herbivore derived- and carnivore derived-*Sarcoptes *mite) among the sympatric wild animals from Asturias.

**Conclusions:**

The confirmation of parasite temporal genetic stability is of vital interest to allow generalizations to be made, which have further implications regarding the genetic structure, epidemiology and monitoring protocols of the ubiquitous *Sarcoptes *mite. This could eventually be applied to other parasite species.

## Background

In the field of parasitology, different molecular markers have been used for parasite genetic characterization and genetic population studies. All molecular studies assume that genetic structure and diversity is relatively stable over time [1,2]. Since allele presence and frequency change over time due to genetic drift, and because of the gene flow between parasite populations from sympatric host species, the assumption of genetic stability may not be accurate [3].

Here we describe, for the first time to our knowledge, a temporal analysis of microsatellite alleles and genetic structure at nine polymorphic loci to examine changes in genetic diversity of *Sarcoptes *mite over time.

*Sarcoptes *mite continues to affect humans and a wide range of mammalian hosts worldwide [4], while the debate about its specificity by the host is still the subject of ongoing debate [5]. An epidemic can result, just from the introduction of a single case of scabies into crowded living conditions [6], which could entail devastating mortality in wild and domestic animals [7,8]. Moreover, recent biochemical and molecular approaches highlighted the threat of emerging acaricide resistance to the treatment of scabies worldwide [9].

*Sarcoptes *mite infections are endemic in many European wild animals and may cause devastating mortality, which has been reported in the Alpine (*Rupicapra rupicapra*) and Pyrenean chamois (*Rupicapra pyrenaica parva*), Iberian ibex *(Capra pyrenaica*), aoudad (*Ammotragus lervia*) and red fox (*Vulpes vulpes*) [10-16]. Notwithstanding, in other sympatric hosts only a few cases have ever been reported such as stone marten (*Martes foina*), badger (*Meles meles*), lynx (*Lynx lynx*), roe deer (*Capreolus capreolus*) and Iberian wolf (*Canis lupus*) [17-19].

Pyrenean chamois (*Rupicapra pyrenaica parva*) population in Asturias (Northern Spain) was affected by a sarcoptic mange epizootic, first detected in 1993. Although the origin of the parasitosis could not be demonstrated, infected domestic goats sharing pastures with wild bovids were suspected to be the source of mites, with subsequent evidence of this cross-infection possibility [20,21]. As reported in epidemics affecting other wild ungulate populations [13-15], *Sarcoptes scabiei *produced an extremely severe effect on chamois population during the first years after eruption [22]. Nowadays the disease can be considered endemic and is still the main health issue affecting Southern chamois.

The number of *Sarcoptes *generations is influenced by the short generation interval, as well as by the infected host's susceptibility and life expectancy, and hence *Sarcoptes *mites on an individual host may in fact form an 'infra-population' [23] that has a number of recurrent generations [24]. *Sarcoptes *population structure is probably that of a species subdivided into genetically small populations with restricted gene flow between local demes [25]. Strong specialisation could be the result of a host taxon-derived shift and, even if two host taxon-derived species are sympatric for their host species, they should be considered as allopatric, if the parasites have no possibility of host choice [26].

The aim of the present study was to test the extent of possible changes in the genetic diversity and structure of *Sarcoptes *mite population from Pyrenean chamois in Asturias within an 11-years interval period (from the epidemic wave in 1997 to the endemic situation in in 2008), and to compare reported molecular data with samples from mangy sympatric red deer, roe deer and red foxes.

## Results

Twenty-nine alleles were detected from the nine microsatellite loci. The allele count for each of the 9 loci ranged from two (Sarms41) to four (Sarms35, Sarms37 and Sarms38). Sixteen private alleles (alleles present in only one population) were detected; all of them were from red fox populations, while no private alleles were detected from the other populations (Table 1). The number of private alleles ranged between one (Sarms34, Sarms36 and Sarms41) and three (Sarms35 and Sarms37).

**Table 1:** Private alleles detected at the 9 microsatellite loci of the red fox-associated mite population, together with their frequencies

Locus	Allele	Frequency
Sarms 33	232	0.5833
	
	240	0.4167

Sarms 34	174	1

Sarms 35	148	0.7222
	
	152	0.1111
	
	156	0.1667

Sarms 36	283	0.6000

Sarms 37	164	0.1667
	
	170	0.5833
	
	178	0.2500

Sarms 38	209	0.4167
	
	211	0.5833

Sarms 40	217	0.7222
	
	243	0.2778

Sarms 41	234	1

Sarms 44	270	0.3636
	
	272	0.0909

The missing data from all the used microsatellite loci was 0.0315, ranging between 0 (for Sarms33, Sarms 37, Sarms38 and Sarms41) and 0.13 (for Sarms36). For all loci examined there was no evidence of LD [linkage disequilibria] (P > 0.05), and no deviation from HWE [Hardy-Weinberg equilibrium] was detected from all loci in all the studied population except Sarms34 and Sarms38 in Pyrenean chamois collected in 1997, and Sarms33, Sarms35-38, and Sarms44 in red fox population.

Allele diversity was identical in all loci from both Pyrenean chamois populations from 1997 and 2008, with the exception of Sarms34 and Sarms38: Sarms34 was monomorphic with only 176 bp allele present in Pyrenean chamois from 1997, while Pyrenean chamois from 2008 has two alleles, 176 bp and 198 bp. The new allele (198 bp) is present in all the other herbivore sympatric populations (red deer and roe deer), but not in the carnivore-derived *Sarcoptes *population (red fox). Sarms38 was monomorphic with only 215 bp allele present in Pyrenean chamois from 1997, while Pyrenean chamois from 2008 has two alleles, 213 bp and 215 bp. Again, the new allele (213 bp) is present in all the other herbivore sympatric populations, but not in the carnivore red fox population.

Intra-host variation was detected in six individuals: one Pyrenean chamois from 1997 (variation in Sarms34), three red deer (variation in Sarms34), and two red foxes (variations in Sarms35 and Sarms40).

AMOVA analysis showed differentiation among populations (*F_ST _*= 0.74808; *P *< 0.001), which indicates that the mite component populations differed greatly. *F_ST _*value between both chamois-derived *Sarcoptes *mite populations was not statically supported (*F_ST _*= 0.1919; *p *= 0.054), while red fox-derived *Sarcoptes *mite population was statistically (*P *< 0.001) different from all other herbivore-derived *Sarcoptes *mite populations (Table 2). These results were confirmed by the average number of pairwise differences between *Sarcoptes *populations: The lowest differentiation was between the two chamois-derived *Sarcoptes *populations, and the highest was between red fox-derived *Sarcoptes *population and the other herbivore-derived *Sarcoptes *mite populations (Table 3). No pairwise differences were detected within *R. pyrenaica *(1997)-derived *Sarcoptes *mite population, while the highest value of the average number of pairwise differences was detected within *V. vulpes*-derived *Sarcoptes *mite population.

**Table 2:** Matrix of significant *F_ST _P *values, with significance level = 0.05 (above diagonal), and population pairwise *F_ST _*(below diagonal) for each pairwise comparison of four *Sarcoptes *mite populations collected in 1997 and 2008 from Asturias, Spain

	*R. pyrenaica-*1997	*R. pyrenaica-*2008	*C. elaphus-*2008	*V. vulpes-*2008
*R. pyrenaica-*1997	-	0.054	< 0.001*	< 0.001*
*R. pyrenaica-*2008	0.1919	-	0.099	< 0.001*
*C. elaphus-*2008	0.4564	0.1179	-	< 0.001*
*V. vulpes-*2008	0.8542	0.7869	0.7556	-

*C. capreolus*-2008 was not included because of the low sampling size.

**Table 3:** Population average pairwise differences between four *Sarcoptes *mite derived populations from Asturias, Spain

	*R. pyrenaica*-1997	*R. pyrenaica*-2008	*C. elaphus*-2008	*V. vulpes*-2008
*R. pyrenaica*-1997	0.00000	0.23077	0.69231	6.91667
*R. pyrenaica*-2008	0.03385	0.39385	0.70118	6.83974
*C.elaphus*-2008	0.27077	0.08272	0.84308	6.91667
*V. vulpes*-2008	5.61957	5.34572	5.19803	2.59420

Above diagonal: Average number of pairwise differences between populations (PiXY). Diagonal elements: Average number of pairwise differences within population (PiX). Below diagonal: Corrected average pairwise difference (PiXY-(PiX+PiY)/2). *C. capreolus*-2008 was not included because of the low sampling size.

**Figure 1:**
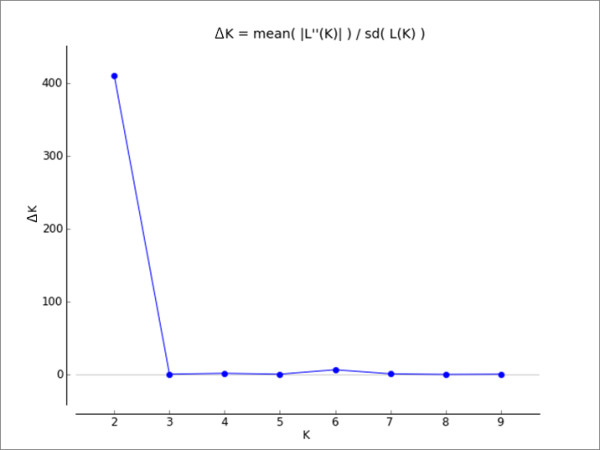
**Results of STRUCTURE analysis showing Δ K as proposed by Evanno et al**. [27]**method**. The best fit of the data was two clusters.

**Figure 2:**
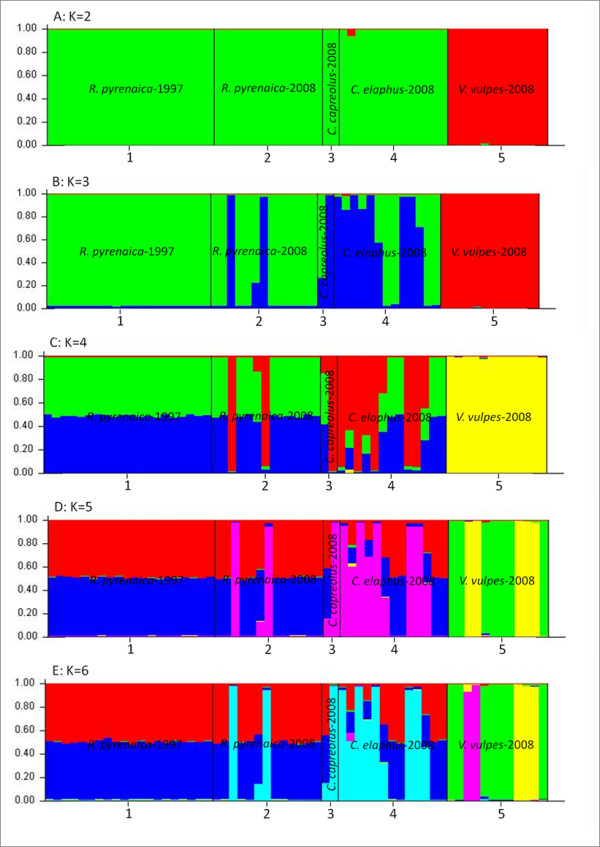
**Bar plotting of the proportion of individual variation of 60 *Sarcoptes *mite from different host species in Asturias (Spain) collected with 11-years interval, assigned to a given genetic clusters in STRUCTURE, when two (A: K = 2), three (B: K = 3), four (C: K = 4), five (D: K = 5), and six (E: K = 6) populations are assumed in the dataset**. Each cluster is represented by a different colour. 1: *R. pyrenaica*-1997. 2: *R. pyrenaica*-2008. 3. *C. capreolus*-2008. 4: *C. elaphus*-2008. 5: *V. vulpes*-2008.

The modal value of the statistic ΔK [27] for the whole dataset showed that the uppermost cluster value was K = 2 (Figure 1). When K = 2, all cluster assignments were consistent with the population of origin. *Sarcoptes *mites from all herbivore hosts were consistently grouped in one cluster, while *Sarcoptes *mite from red fox (carnivore host) formed another well-supported cluster (Figure 2).

The posterior probability analyses supported our results, since we obtained similar grouping when applying K = 3, 4, 5 and 6, which demonstrates the complete resolution of populations into distinct clusters (herbivore- and carnivore-derived *Sarcoptes *mite populations), and illustrates the subpopulations within *R. pyrenaica*-2008- and *V. vulpes*-derived *Sarcoptes *mite populations (Figure 2).

**Figure 3:**
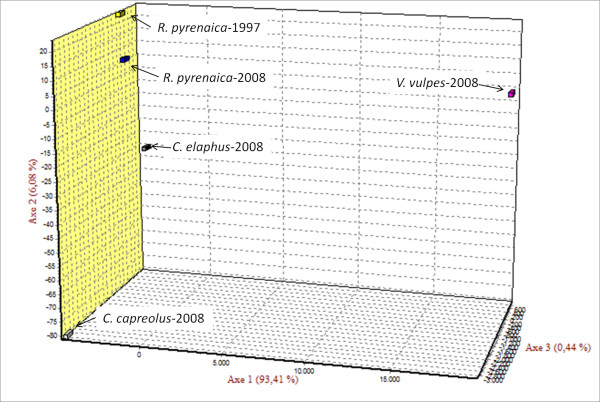
**Factorial Component Analysis (FCA) of the proportion of variation of five *Sarcoptes *mite populations from Asturias (Spain) assigned to a given genetic clusters in Genetix**.

The scatter plot of the FCA, for individuals (data not shown) and populations (Figure 3) of the microsatellite genotypes using *Sarcoptes *mite collected from the sympatric wild animals from Asturias, confirmed the results obtained by the Bayesian assignment test. The two chamois-derived *Sarcoptes *populations were similar and close to the other herbivore-derived populations (red deer and roe deer), and well-differentiated from the carnivore (red fox)-derived *Sarcoptes *population.

## Discussion

As with other highly divergent taxa, with *Sarcoptes scabiei *few loci and low sample sizes are sufficient to find strong population differentiation between host species [28,29]. The unusually high number of private alleles in red fox population, was the first indicator of the genetic separation and lack of gene flow between *Sarcoptes *mite from this carnivore animal and the sympatric herbivores (roe deer, red deer and Pyrenean chamois), which is in concordance with the host-taxon effect among *Sarcoptes *populations from different sympatric wild animals [26].

The few detected cases of infra-host variations could be attributed to the skin-scale phenomenon [24], while the deviation from HWE presented in some loci from the red fox population could be attributed to possible subpopulations within *Sarcoptes *mites from this host [30,31]. *Sarcoptes *mites lack free-living stages, and individual hosts, depending on their susceptibility and behaviour, are essentially ephemeral habitats providing patchy environments that hamper random mating [32,33].

Two new alleles were detected in two different loci from the 2008-Pyrenean chamois population comparing with the 1997-population. Both new alleles are present in all the other herbivore sympatric populations, but not in the carnivore red fox population. This could be understood as small change in allele diversity within chamois-derived *Sarcoptes *population during the 11-years interval period, which could be attributed, simply to non-random sampling, or to little gene flow from the other sympatric herbivore(red deer and roe deer)-derived *Sarcoptes *mite, (since our first 1997-sampling coincided with the start of *Sarcoptes *outbreak wave in chamois, and it is possible that more waves of *Sarcoptes *transmission with the other sympatric herbivore hosts have took place until the current endemic situation of *Sarcoptes *mite in this 2008-chamois population) but never from the carnivore sympatric host (red fox), following the host-taxon law [26].

All AMOVA analysis (showing differentiation among populations), the Bayesian assignment test (between mites), and the scatter plot of the FCA (for individuals and populations) confirmed the absence of genetic differentiation between the two chamois-derived *Sarcoptes *mite populations collected in 11-year interval period. On the other hand, the results corroborate the presence of a host-taxon phenomenon and lack of gene flow or recent admixture between carnivore- and herbivore-derived *Sarcoptes *populations, among *Sarcoptes *mites from wild animals in Asturias, in concordance with other European wild hosts [26].

Mite transmission may occur within each host taxon-derived *Sarcoptes *mite population (explaining temporal and geographical coincidences reported between chamois and red deer sarcoptic mange cases in the studied area and confirming their suspected common origin; [34]), but it seems to be extremely rare or absent between them [26].

## Conclusions

The analysis of 11-year interval period found little change in the genetic diversity and showed clear temporal stability in the genetic structure of *Sarcoptes *mite population under the host-taxon law. The understanding of this factor is crucial, if generalizations are to be made concerning temporal genetic stability. Besides the genetic implications of our results, this study could have further ramification in the epidemiological studies and the monitoring protocols of the neglected *Sarcoptes *mite, and could have further applications in other parasite species.

## Methods

### Specimen collection and DNA extraction

Using postponed isolation and direct isolation (with aqueous potassium hydroxide digestion) techniques [35] sixty representative adult mites were collected during two different periods, 1997 and 2008: (i) in 1997, twenty *Sarcoptes *mite were collected from the skin crust of ten infected Pyrenean chamois, and (ii) in 2008, fourteen parasites were collected from the skin crust of seven infected Pyrenean chamois, two mites from two mangy roe deer, thirteen from eight infected red deer, and twelve from six red fox.

All mites were identified as *S. scabiei *on the basis of known morphological criteria [36]. The DNA of individual *Sarcoptes *mites was extracted using the HotSHOT Plus ThermalSHOCK technique [37], as following: 25 μl of an alkaline lysis reagent (25 mM NaOH, 0.2 mM disodium EDTA; pH = 12) was used as a substrate for individual *Sarcoptes *mite DNA extraction by three cycles of thermal shock (2 min at -80°C, freezing step, and 15 s at +70°C, thawing step), followed by a short incubation (30 min at 95°C) and pH adjustment with 25 μl of a neutralizing reagent (40 mM Tris-HCl; pH = 5). Two blanks (reagents only) were included in each extraction to monitor for contamination.

### Fluorescent-based polymerase chain reaction analysis of microsatellite DNA

As described by Alasaad et al. [24], nine specific *Sarcoptes *mite microsatellites (Sarms 33-38, 40, 41 and 44) were used with one 9× multiplex PCR. One primer from each set was 5' labelled with 6-FAM, VIC, NED or PET^® ^fluorescent dye tag (Applied Biosystems, Foster City, CA, USA). Each 15 μl PCR reaction mixture consisted of 3 μl of the single mite DNA, together with the PCR mixture containing all primer pairs (ranged from 0.04 to 0.1 μM per primer), 200 μM of each dNTP, 1.5 μl of 10× PCR buffer (200 mM KCl and 100 mM Tris-HCl, pH 8.0), 1.5 mM MgCl2 and 0.15 μl (0.5 U/reaction) HotStar Taq (QIAGEN, Milano, Italy). The thermal profile in a 2720 thermal cycler (Applied Biosystems, Foster City, CA, USA) was as following: 15 min at 95°C (initial denaturing), followed by 37 cycles of three steps of 30 s at 94°C (denaturation), 45 s at 55°C (annealing) and 1.5 min at 72°C (extension), before a final elongation of 7 min at 72°C. Fluorescent PCR amplification products were analyzed using formamide with Size Standard 500 Liz (Applied Biosystems, Foster City, CA, USA) by ABI PRISM 310 Genetic Analyzer with pop4. Allele calling was performed using the GeneMapper v. 4.0 software (Applied Biosystems, Foster City, CA, USA).

### Molecular analyses

Expected (*H_E_*) and observed (*H_O_*) heterozygosity, linkage disequilibria (LD), and Hardy-Weinberg equilibrium (HWE) tests were calculated using GENEPOP (v.3.4; [38]). Deviations from HWE and tests for LD were evaluated using Fisher's exact tests and sequential Bonferroni corrections. We estimated genetic diversity using three values; mean number of alleles, expected, and observed heterozygosity based on data from all nine loci. Possible genotyping mistakes (scoring error due to stuttering, large allele dropout) were estimated using MICROCHECKER [39].

The heterogeneity of genetic diversity among the different *Sarcoptes *mite populations was estimated by the partition of variance components (AMOVA) applying conventional *F_ST _*statistics using allele frequencies as implemented in Arlequin 3.11 [40]. The analysis of relationships between mites was carried out by the Bayesian assignment test of the software STRUCTURE (v.2.3.3; [41]). Burn-in and run lengths of Markov chains were both 100000. We ran 30 independent runs for each K (for K = 1-10). The most likely number of clusters was determined using the method of Evanno et al. [27]. Finally, each of the inferred clusters was associated with the component populations of its mites.

The degree of genetic relationship among populations was further investigated with FCA (Factorial Component Analysis) as implemented in Genetix v.4.05.2 [42].

## Competing interests

The authors declare that they have no competing interests.

## Authors' contributions

CG, RC, AO, LR and RCS conceived and designed the experiments. AO, RC and CG performed the field work experiments. AMM, AO and SA performed the molecular work. Manuscript was written by all co-authors. All authors read and approved the final manuscript.
